# *In Vitro* Analysis of Bacterial Microcompartments and Shell Protein Superstructures by Confocal Microscopy

**DOI:** 10.1128/spectrum.03357-22

**Published:** 2023-02-14

**Authors:** Daniel S. Trettel, Wade C. Winkler

**Affiliations:** a Department of Chemistry and Biochemistry, The University of Maryland, College Park, College Park, Maryland, USA; b Department of Cell Biology and Molecular Genetics, The University of Maryland, College Park, College Park, Maryland, USA; University of Minnesota—Twin Cities

**Keywords:** bacterial organelles, microcompartments, confocal microscopy

## Abstract

The shell proteins that comprise bacterial microcompartments (BMCs) can self-assemble into an array of superstructures such as nanotubes, flat sheets, and icosahedra. The physical characterization of BMCs and these superstructures typically relies on electron microscopy, which decouples samples from their solution context. We hypothesize that an investigation of fluorescently tagged BMCs and shell protein superstructures *in vitro* using high-resolution confocal microscopy will lead to new insights into the solution behavior of these entities. We find that confocal imaging is able to capture nanotubes and sheets previously reported by transmission electron microscopy (TEM). Using a combination of fluorescent tags, we present qualitative evidence that these structures intermix with one another in a hetero- and homotypic fashion. Complete BMCs are also able to accomplish intermixing as evidenced by colocalization data. Finally, a simple colocalization experiment suggests that fluorescently modified encapsulation peptides (EPs) may prefer certain shell protein binding partners. Together, these data demonstrate that high-resolution confocal microscopy is a powerful tool for investigating microcompartment-related structures *in vitro*, particularly for colocalization analyses. These results also support the notion that BMCs may intermix protein components, presumably from the outer shell.

**IMPORTANCE** Microcompartments are large, organelle-like structures that help bacteria catabolize targeted metabolites while also protecting the cytosol against highly reactive metabolic intermediates. Their protein shell self-assembles into a polyhedral structure of approximately 100 to 200 nm in diameter. Inside the shell are thousands of copies of cargo enzymes, which are responsible for a specific metabolic pathway. While different approaches have revealed high-resolution structures of individual microcompartment proteins, it is less clear how these factors self-assemble to form the full native structure. In this study, we show that laser scanning confocal microscopy can be used to study microcompartment proteins. We find that this approach allows researchers to investigate the interactions and potential exchange of shell protein subunits in solution. From this, we conclude that confocal microscopy offers advantages for studying the *in vitro* structures of other microcompartments as well as carboxysomes and other bacterial organelles.

## INTRODUCTION

Bacterial microcompartments (BMCs) are a widely distributed class of prokaryotic organelles (1–3). These subcellular structures are typically 40 to 300 nm in diameter and are composed entirely of protein. The protein elements are segregated into a distinct icosahedral protein shell and its inner enzymatic cargo (1, 4). The inner cargo is responsible for a diverse range of chemistries (2, 3, 5), from carbon fixation (6) to glycyl radical enzyme mechanisms (7, 8). The overall modular arrangement of BMCs has led researchers to propose repurposing BMCs into an accessible platform for synthetic biology efforts (4, 9–11). However, synthetic adaptations of BMCs have generally been slowed by inadequate knowledge of the “rules” that govern BMC assembly.

The BMC protein shells self-assemble into a honeycomb-like lattice that selectively gates the influx and efflux of key metabolites. Specifically, the facets of the shells are made from a combination of flat, cyclic homohexamers (BMC-H) and homotrimers (BMC-T), and the vertices are capped by similarly arranged pentamers (BMC-P) (12, 13). Recently, high-resolution cryo-electron microscopy experiments have captured exquisite details of synthetic BMC shells, providing important clues into the way that the shell “tiles” piece together (14–17). While these synthetic shells are small (typically <40 nm in diameter), larger synthetic BMC shell platforms have also been reported (18, 19).

Individual shell proteins are not capable of polymerizing into the polyhedral BMC structure; instead, combinations of different classes of shell proteins are thought to be required for this to occur. This implies that there are intrinsic physicochemical properties of the different classes of shell proteins that guide their successful polymerization into polyhedral structures. Interestingly, in the absence of the correct partners, some of the individual shell proteins have been reported to form alternate superstructures. For example, a few BMC-H proteins have been noted to self-assemble into tube-like structures (coined “nanotubes”) when purified *in vitro* (20–24). Nanotubes have also been reported for certain BMC-T proteins (21), although BMC-P types have not been associated with these structures. These purified nanotubes were observed by electron microscopy in bundles or as individual tubes (21, 24). Bundles of BMC-H nanotubes may even be capable of forming within bacterial cells upon the overexpression of BMC-H proteins (19, 20, 23, 24). Furthermore, these BMC-H nanotube bundles appear to interfere with septation, thereby resulting in elongated and linked cells (23).

These nanotube structures have not been systematically investigated or analyzed by solution-based experimental approaches. It is not known whether they are still capable of interacting with other shell proteins or with cargo proteins. Therefore, a deeper investigation of these remarkable structures will give new and important insight into the protein-protein interactions that occur within BMCs. It would also potentially lead to new synthetic biology tools (25) warranting considerable further study.

Here, we apply multiple imaging techniques to investigate the structures formed by BMC shell proteins *in vitro*. We chose to analyze well-characterized shell proteins used by the Salmonella enterica propanediol utilization (Pdu) BMC. The Pdu BMC is composed of approximately 8 shell proteins (PduABB′JKNTU) as well as an encapsulated signature diol dehydratase (PduCDE), an aldehyde dehydrogenase (PduP), and an alcohol dehydrogenase (PduQ), among others. We use laser scanning confocal microscopy to analyze several major shell proteins (PduABB′J) and their associated superstructures, including native BMCs. As a solution technique, confocal microscopy further allows colocalization events to be observed when different shell proteins are equilibrated, suggesting the intermixing of components. When preparations of BMCs that had been tagged with different fluorophores were mixed, we found that these fluorophore-tagged proteins also exhibited increasing colocalization over time. Consistent with this, we find that the native BMC can associate with the nanotubes. Finally, we showcase another utility of this mode of analysis to directly show that encapsulation peptides (EPs), which are known to target cargo proteins to the inner lumen of the BMC, associate with certain shell protein structures. Together, our data give proof-of-principle evidence that confocal microscopy-based approaches can be used to study BMC-related structures in solution, including their propensity to exchange protein subunits.

## RESULTS

### The hexamer PduA forms nanotubes that can be imaged by confocal microscopy.

BMCs and nanotubes formed from BMC shell proteins have been previously investigated by transmission electron microscopy (TEM) (21, 23, 24). While TEM remains a powerful tool for analyzing BMC-related structures, we reasoned that BMC proteins might also be imaged using laser scanning confocal microscopy if the proteins were fluorescently labeled. While suffering from lower resolution, this approach would offer several practical advantages over electron microscopy. For example, the treatment of BMC samples for electron microscopy can lead to the deformation or alteration of structural morphology (26). In contrast, BMCs and BMC proteins imaged by confocal microscopy can remain in aqueous solution under standard buffer conditions. Also, multiple fluorophores can be utilized for confocal microscopy, thereby allowing the simultaneous imaging of multiple BMC factors. This would allow investigations of the interactions of BMC proteins.

**FIG 1:**
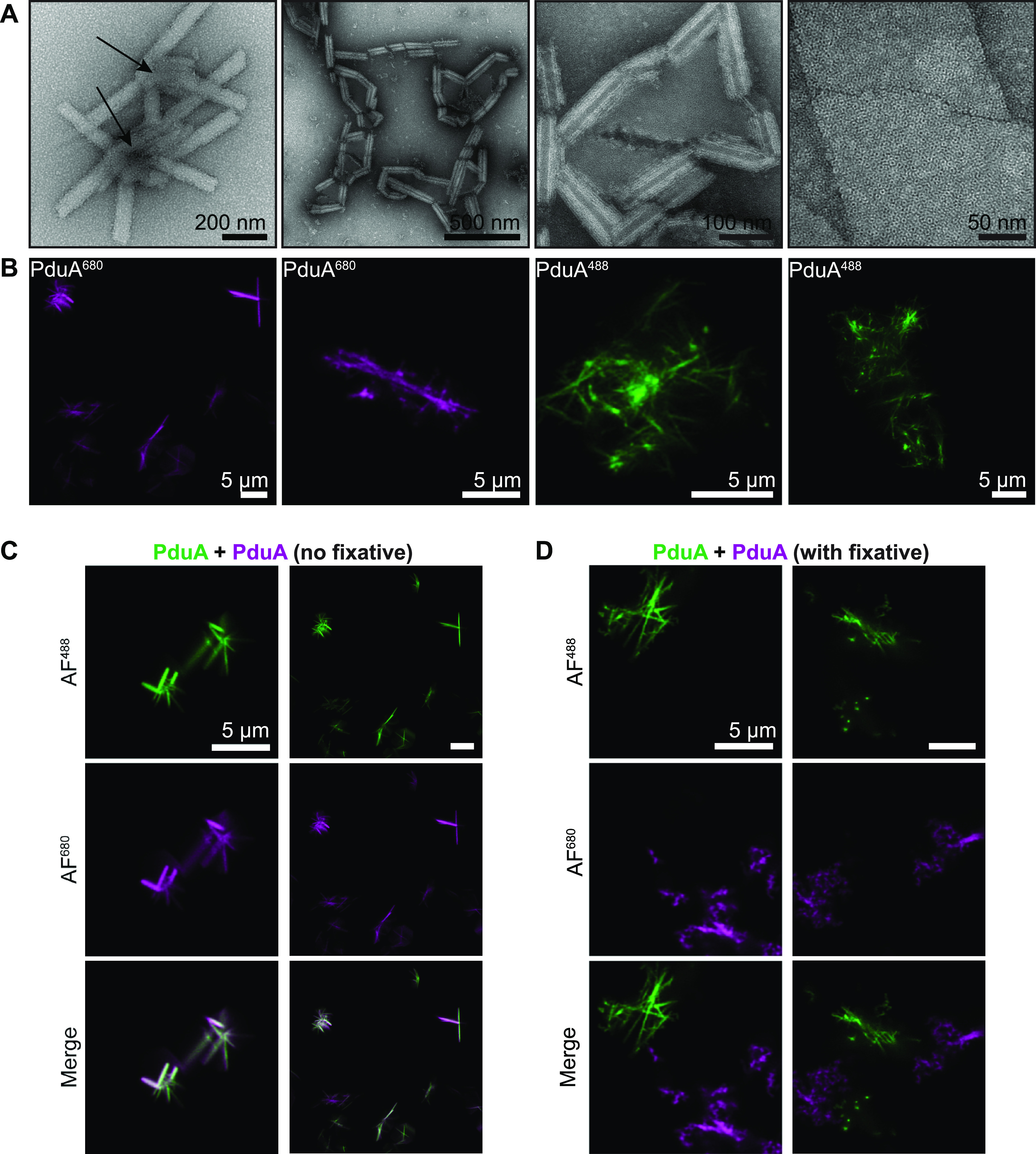
Imaging of PduA superstructures. (A) Transmission electron microscopy (TEM) analysis of PduA structures reveals clusters of nanotubes. Arrows denote clustering centers. (B) Laser scanning confocal microscopy analysis of Alexa Fluor 680-tagged PduA (PduA^680^) reveals protein nanotubes. (C) PduA samples labeled with different fluorophores were mixed and equilibrated to observe their colocalization. (D) A similar set of aliquots was fixed with paraformaldehyde prior to incubation, preventing their colocalization.

The BMC-H protein PduA, which is from the Salmonella enterica propanediol utilization (Pdu) BMC, has previously been reported to form nanotubes when purified to homogeneity (19, 21, 23, 24). To test whether PduA nanotubes could be imaged using confocal microscopy, we purified a conserved S93C mutant of PduA (referred to as just PduA here). The S93C mutation was previously shown to enable the site-specific modification of PduA with a maleimide-conjugated fluorophore (27). PduA was successfully purified to homogeneity and fluorescently labeled, as determined by SDS-PAGE (see Fig. S1 in the supplemental material). TEM analysis of purified, labeled PduA confirmed its previously reported ability to form a series of superstructures, including nanotubes and sheets (Fig. 1A). Interestingly, some nanotubes are found in clusters around a central site (Fig. 1A, arrows). In contrast, we did not observe bundles of PduA nanotubes, as was previously reported. This difference may stem from the use of different mutants of PduA and different buffer conditions, critical parameters, compared to those in previous reports (21).

Next, we turned to the imaging of the PduA structures with a confocal microscope equipped with an Airyscan detector for enhanced resolution (28). Under these conditions, PduA presents as dispersed clusters of objects that appear, roughly, like nanotubes (Fig. 1B). These clusters are several micrometers in length and could also be observed under bright-field conditions (Fig. S2). We find that these structures are agnostic to the fluorescent modification: filamentous structures were observed in samples labeled with both Alexa Fluor 680 (AF^680^) and AF^488^. More detail could be resolved with AF^488^-labeled samples due to the shorter wavelengths that are used for imaging. These results show that BMC-H nanotubes can be studied *in vitro* with a confocal microscope.

Confocal analysis can discriminate between different objects if they are labeled with different fluorophores. Given this, we sought to explore if separate sets of PduA would colocalize if equilibrated. To investigate this, separate aliquots of AF^488^- and AF^680^-labeled PduA were mixed and equilibrated for 30 min. A control where the aliquots were fixed with paraformaldehyde prior to mixing was also analyzed. Confocal images were then taken at the end of the equilibration period. This revealed evidence of colocalization for the two fluorophores, suggesting that PduA superstructures may equilibrate subunits (Fig. 1C). In contrast, fluorescence signals for fixed samples remained separate (Fig. 1D). These results imply that individual PduA hexamers may equilibrate between soluble hexamer and bound nanotube forms. Fixation prevents this equilibration from occurring, resulting in no exchange between separate superstructures. While we demonstrate only endpoint results, these data argue that laser scanning confocal microscopy can indeed be utilized as an experimental approach for the real-time imaging of nanotubes.

### The hexamer PduJ forms distinct nanotubes.

**FIG 2:**
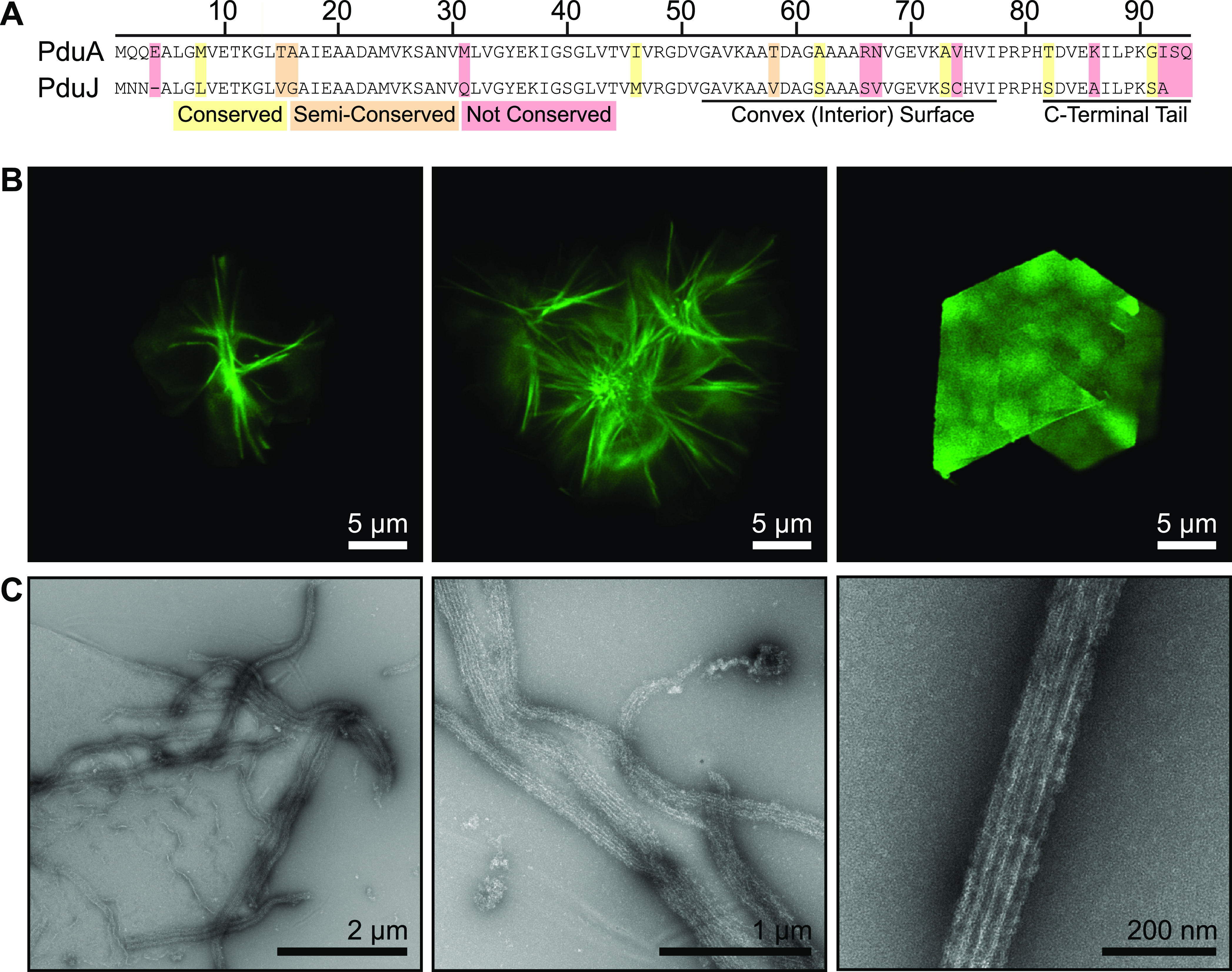
Imaging of PduJ superstructures. (A) Sequencing alignment of S. enterica PduA and PduJ showing how similar/identical these two structural components are. Alignment was performed using Clustal X2. (B) Laser scanning confocal microscopy analysis of Alexa Fluor 488-tagged PduJ reveals protein rosettes of nanotubes and large sheet structures. (C) Transmission electron microscopy (TEM) of PduJ shows that rosettes are likely to be interweaving clusters of flexible nanotubes.

The PduJ shell protein has also recently been reported to form nanotubes (23). The sequence of PduJ closely resembles that of PduA, with a high degree of sequence identity across the lengths of the proteins (Fig. 2A) (29). PduA and PduJ behave similarly enough that many consider them to be interchangeable, redundant, and functionally identical to one another (23, 29, 30). Therefore, we sought to determine whether PduJ could form nanotubes akin to PduA. However, while our data revealed that fluorophore-labeled PduJ indeed formed nanotubes, these structures exhibited morphologies distinct from those of PduA. Under our assay conditions, PduJ presented as large, rosette-like structures rather than individual nanotubes (Fig. 2B; Fig. S2). Based on this observation, we speculate that the PduJ rosettes might have a complex internal structure. Like PduA, PduJ can also form sheet structures (Fig. 2B, right). Interestingly, the higher resolution offered by TEM presented us with images that appeared to suggest that the rosettes consist of a network of flexible, interweaving nanotubes (Fig. 2C). This flexibility has not previously been noted for BMC-H nanotubes. Together, these data show that PduJ can form nanotubes but that these structures are different from PduA in terms of apparent flexibility despite the close sequence homology of these proteins. The sequence differences that do exist, however, lie predominantly along the convex surface, which likely dictates cargo interactions, and along their C-terminal tails. These terminal tails have been suggested to be disordered, dynamic, and potentially responsible for dictating interactions between adjacent shell proteins (27, 31). However, from these data alone, we cannot offer a definitive mechanistic explanation for the structural differences presented here, although we note that confocal microscopy offers a straightforward approach for investigating superstructures formed by PduA/J proteins containing site-directed mutations.

### Different shell proteins can colocalize.

We reasoned that confocal microscopy could provide valuable insight into the solution behaviors of the PduA and PduJ structures if different shell proteins, carrying different fluorescent tags, were equilibrated and observed as in Fig. 1C and D. This would, for example, allow us to investigate whether different nanotubes might be able to measurably exchange protein subunits. For these experiments, we also purified the major BMC-T proteins PduB and PduB′ (an alternative translation product of the *pduB* gene that lacks the N-terminal 37 residues). After their purification, we found that both PduB and PduB′ produce aggregates in solution (Fig. S3). However, PduB forms significantly larger aggregates than PduB′, suggesting that the N-terminal sequence enhances the propensity to aggregate. Previously reported PduB nanotubes (from Lactobacillus reuteri DSM20016) were not observed under our assay conditions (21). However, another study using PduB from S. enterica, as we do here, likewise found a lack of evidence of PduB-derived nanotubes with *in vivo* tests (23).

**FIG 3:**
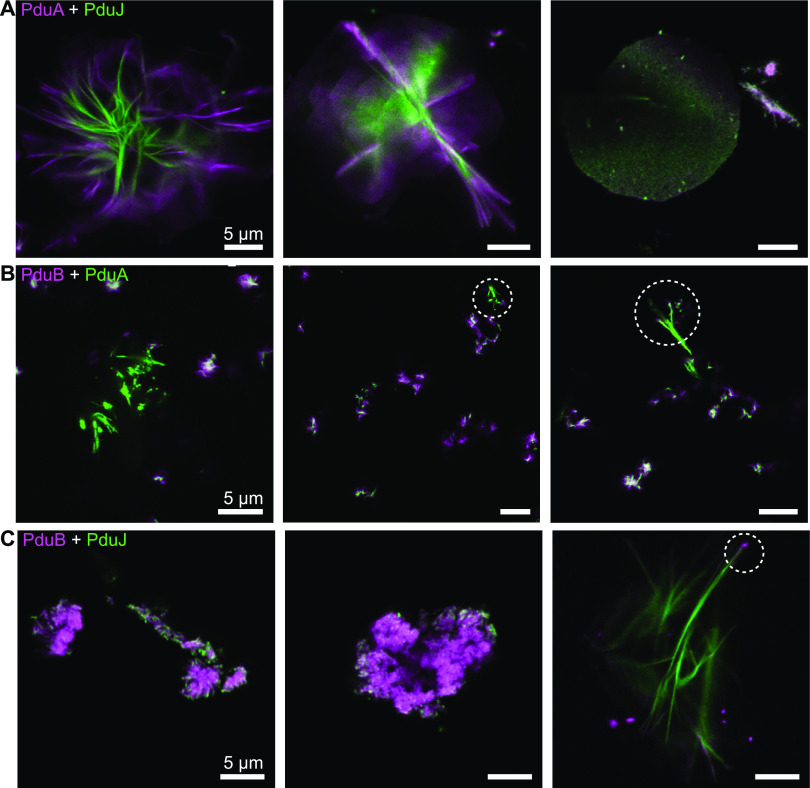
Confocal microscopy imaging of mixtures of shell proteins reveals the exchange of subunits. (A) Alexa Fluor 680-tagged PduA was equilibrated with Alexa Fluor 488-labeled PduJ and then imaged by laser scanning confocal microscopy. Both rosettes and sheets were identified by microscopy; they featured a colocalized mixture of PduA and PduJ proteins. (B) Confocal microscopy imaging of PduA mixed with PduB. PduA nanotubes exhibited colocalization of PduB at or near their poles. PduA showed general colocalization with PduB aggregates. (C) Confocal microscopy imaging of PduJ mixed with PduB. PduJ appears to associate with the periphery of PduB aggregates, while PduB appears to associate at the poles of PduJ nanotubes. Circles indicate the colocalization of PduB to nanotube poles.

PduA and PduJ, each carrying a distinct fluorescent tag, were mixed and equilibrated on ice for 30 min prior to imaging. Confocal analysis revealed that subunits of the PduA and PduJ structures could colocalize albeit with interesting caveats. For instance, the imaging of rosette structures, typically associated with just PduJ, showed an interesting molecular phenotype for the mixtures of PduA and PduJ. We found that the PduA/PduJ structures were hybrid, displaying a PduJ core with a periphery that featured PduA subunits (Fig. 3A). From this, we speculate that PduA might equilibrate initially with the exterior of the PduJ rosettes before permeabilizing inward into the PduJ superstructure. Importantly, these results reflect not only that PduA and PduJ interact but also that some or all of their respective structures can equilibrate.

Next, we tested whether we could observe interactions of PduA and PduJ with PduB and PduB′. PduB and, by extension, PduB′ were previously suggested to interact with PduA (19, 32) and, more recently, PduJ (27). Therefore, we again employed confocal imaging to directly investigate the colocalization of PduB/PduB′ proteins with PduA and PduJ superstructures. This showed that the mixing of PduA and PduB led to a combination of their two independent morphologies (i.e., nanotubes plus aggregates). However, the colocalization trends differed between the PduA nanotubes and the PduB aggregates (Fig. 3B). PduA nanotubes displayed nearly no overlap of the PduB signal except for at the end of nanotubes (Fig. 3B, circles). Conversely, it was not uncommon for PduB aggregates to display colocalization with the PduA signal, although further work is needed before quantitative statements can be made about putative PduB-PduA interactions in this context. We observed similar findings for the equilibration of PduB with PduJ (Fig. 3C) as well as for PduB′ (Fig. S3C and D). Together, these results tentatively confirm that PduB interacts with both PduA and PduJ and suggest that individual protein subunits might be exchanged in characteristic ways for the respective PduABB′J structures. Although more work is required for quantitative statements, our data also suggest that PduB can only colocalize to the ends of PduAJ nanotubes rather than extend from them, as we observed with the PduAJ mixtures. This is consistent with our finding that PduBB′ are unable to extend/form their own nanotubes.

### Pdu BMCs can colocalize *in vitro*.

**FIG 4:**
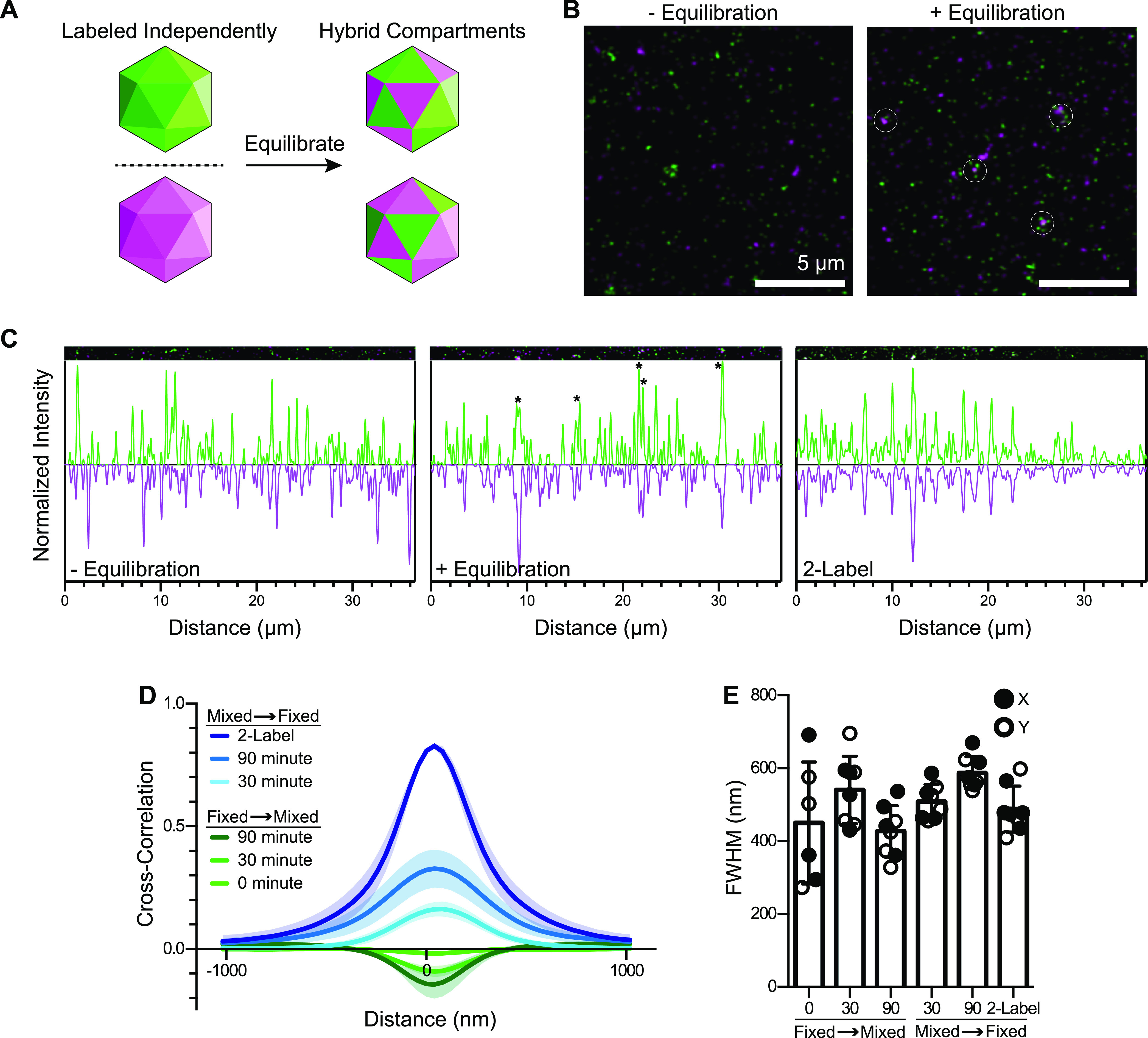
Proteins from Pdu BMCs can colocalize after equilibration. (A) Schematic of the experimental design. Separately labeled BMC batches (Alexa Fluor 680 and Alex Fluor 488) were mixed, equilibrated, and imaged by laser scanning confocal microscopy. (B) To examine whether BMC preparations exhibited colocalization of BMC factors, samples either were imaged by confocal microscopy without being equilibrated or were equilibrated for 30 min on ice before being imaged. Equilibrated samples revealed the presence of white foci (circled). (C) Plotting signal intensities from slices of micrographs. Colocalization events could be detected for equilibrated samples and for positive-control samples that had been labeled with both fluorophores. (D) Pdu BMCs were fixed either before (“Fixed → Mixed”) or after (“Mixed → Fixed”) being allowed to equilibrate, imaged, and analyzed for cross-correlations via the van Steensel method (33). Four micrographs for each sample were analyzed in both the *x* and *y* directions. (E) Plots of the full widths of the Gaussian distributions in panel D at half-maximum (FWHM), showing that particle size estimates appear approximately equal for all samples.

Native BMCs can be simplistically regarded as a specific collection of shell protein components. Accordingly, we believed that native Pdu BMCs may act similarly to individual shell proteins regarding their ability to colocalize. In previously reported experiments, we purified fully formed Pdu BMCs and showed that they can be reacted with different chemical probes (i.e., fluorophores and cross-linkers) (27). Therefore, we again purified Pdu BMCs and tagged aliquots with different fluorophores (Fig. S4A). TEM analysis of treated samples confirmed that they maintained a morphology similar to that of untreated Pdu BMCs (Fig. S4B), suggesting that minimal sample damage had been incurred by the chemical probes. We then mixed equimolar amounts of the two BMC solutions and allowed them to equilibrate (Fig. 4A). After aliquots of these mixtures were removed at short and longer time points, they were fixed with paraformaldehyde and a cross-linker and then analyzed by laser scanning confocal microscopy.

We began by comparing differently labeled batches of Pdu BMCs that either were fixed prior to equilibration and imaging (as a negative control) or were allowed to equilibrate prior to imaging (Fig. 4B). The samples that were fixed prior to being mixed appeared as distinct, distinguishable foci (shown as either green or magenta), showing no visual evidence of colocalization, likely owing to the large extent of fixation achieved (Fig. S4C). In contrast, the samples that were mixed and equilibrated prior to the addition of the fixative showed increasing evidence of colocalization over time. A sample of BMCs that were deliberately dually labeled by both fluorophores was also developed as a positive colocalization control. To analyze these data, we quantified slices of micrographs from nonequilibrated, equilibrated, and dually labeled samples and independently plotted the intensities of the green and magenta signals (Fig. 4C). The samples that were fixed before being mixed showed an apparent random peak distribution, showing no evidence of colocalization. In contrast, the dually labeled sample, as anticipated, showed a high degree of overlap between signal peaks, signifying the colocalization of the two fluorophores. Importantly, the samples that were mixed and equilibrated showed colocalization of the two types of signal peaks.

We next turned to van Steensel cross-correlation analysis to gather deeper insight into focus colocalization in a less biased manner. Here, cross-correlation is measured as a function of a pixel shift (i.e., distance) between the green and magenta scans (33, 34). The resulting Gaussian curve can be positive (indicating correlation), near zero (no correlation), or negative (anticorrelation). Pdu BMC samples that were fixed and then equilibrated for either 0, 30, or 90 min prior to imaging all displayed modest anticorrelations (Fig. 4D and E). The dually labeled BMC positive control, in contrast, displayed an extremely high cross-correlation value of 0.85. Samples that were equilibrated for 30 or 90 min prior to imaging showed positive cross-correlations of 0.18 and 0.33, respectively. van Steensel analysis also yields data on particle sizes (the full width at half-maximum for the curve), which were found to be similar in all samples, thus indicating that sample treatment is not majorly disruptive (Fig. 4F), in agreement with the TEM results (Fig. S4B). Together, these data support the hypothesis that fully formed, native Pdu BMCs can be examined by laser scanning confocal microscopy and, furthermore, may be capable of exchanging some of their components.

### Pdu BMCs interact with shell protein superstructures.

**FIG 5:**
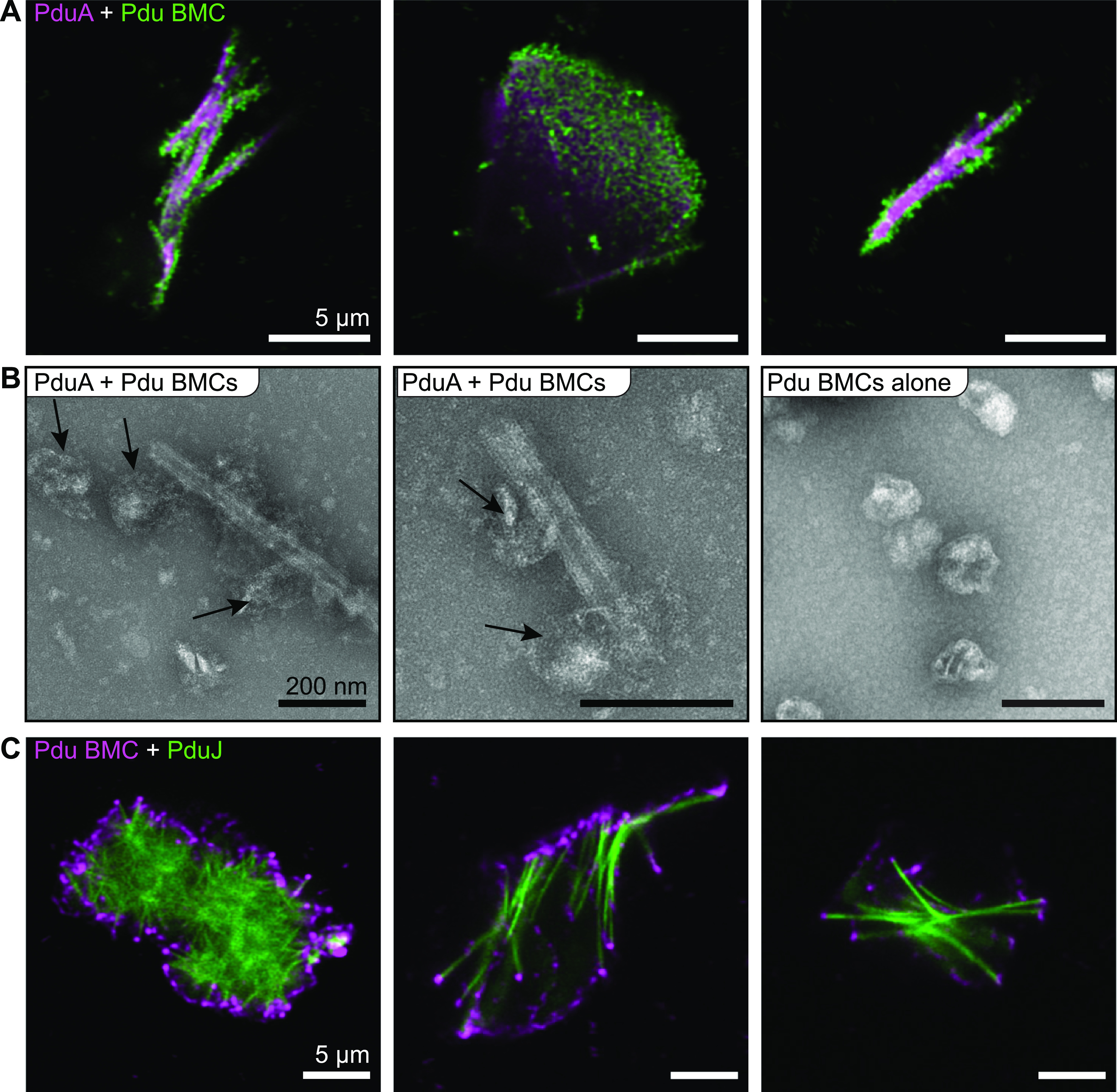
Full-sized, native Pdu BMCs interact with shell protein superstructures. (A) Alexa Fluor 680-tagged PduA nanotubes were equilibrated for 30 min on ice with Alexa Fluor 488-tagged Pdu BMCs and then imaged by laser scanning confocal microscopy. BMCs appear to colocalize along PduA superstructures. (B) Samples from panel A were analyzed by transmission electron microscopy (TEM). BMCs (arrows) stick to and encompass PduA nanotubes but appear to show a loss of overall structural integrity. Pdu BMCs (right) serve as a morphology control. (C) Alexa Fluor 488-tagged PduJ was equilibrated for 30 min on ice with Alexa Fluor 680-tagged Pdu BMCs and then imaged by laser scanning confocal microscopy. BMCs appear to colocalize with PduJ superstructures in characteristic ways.

The results reported here have so far suggested that both the superstructures of Pdu shell proteins and the fully formed Pdu BMCs are capable of exchanging protein constituents. We speculate that this exchange occurs through an equilibrium that is established between the complete nanotubes/polyhedra and their individual shell proteins in solution. Here, Pdu BMCs can be roughly modeled as a specific collection of shell elements much like the nanotubes. Accordingly, we reasoned that the mixing of the protein nanotubes with Pdu BMCs might also result in the exchange of protein subunits or even hybrid structures, which could again be observable via confocal microscopy. We first tested this by equilibrating labeled PduA nanotubes with labeled Pdu BMCs and examining the mixtures using laser scanning confocal microscopy (Fig. 5A). Unexpectedly, this revealed striking images of PduA nanotubes featuring BMC foci dotted along their surface (Fig. 5A). Similarly, PduA sheets also appeared to be speckled by BMC foci. To investigate the BMC foci further, we analyzed the PduA-BMC mixtures by TEM. This revealed the presence of large BMC-like entities annealed to PduA nanotubes (Fig. 5B). However, these BMC structures appeared to be much less morphologically defined than in the absence of nanotubes (Fig. 5B, right). In fact, the BMC structures appeared to envelop the PduA nanotubes in a manner that leaves the BMC entities partially deformed (Fig. 5B). Similarly, we found that Pdu BMCs also adhered to the exterior of the PduJ rosettes in solution (Fig. 5C), although this was not investigated further by TEM.

### Encapsulation peptides display a preference for shell proteins.

To further demonstrate the potential utility of confocal microscopy for the analysis of BMC proteins, we opted to directly visualize the interactions of shell superstructures with encapsulation peptides (EPs). Cargo proteins are generally thought to interact with shell proteins and each other via short helical termini called EPs. In the Pdu BMC system, the EPs for the cargo proteins PduD and PduP have both been proposed to interact with PduA, possibly at its C terminus (35, 36). Furthermore, the EP of PduP has also been suggested to interact with another BMC-H protein, PduK (37). In separate studies, the N terminus of PduB has been speculated to act as an anchor between the inner cargo and the shell (27, 38). If true, we reasoned that purified EPs may associate directly with some or all shell protein superstructures, which could be observed as colocalization events.

**FIG 6:**
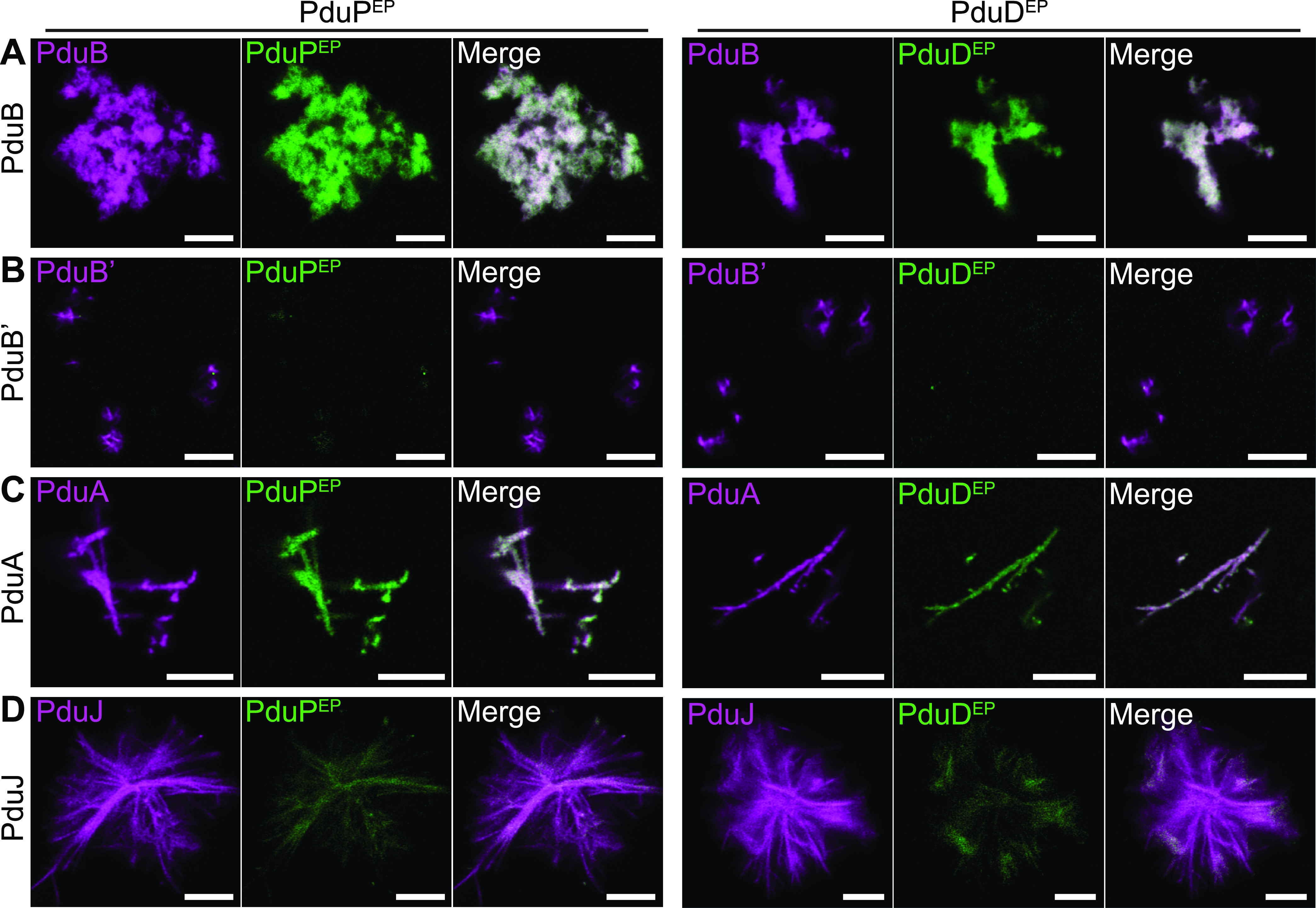
Association of encapsulation peptides with shell protein superstructures. Purified, synthetic, fluorescein-tagged encapsulation peptides (EPs) for PduP (PduP^EP^) and PduD (PduD^EP^) were equilibrated with either PduB (A), PduB′ (B), PduA (C), or PduJ (D). In all instances, samples were equilibrated for 30 min on ice before being imaged by laser scanning confocal microscopy. Both EPs behaved similarly; a higher degree of colocalization was observed between the EPs and PduB and PduA. Bars, 5 μm.

The EPs for PduD and PduP (PduD^EP^ and PduP^EP^, respectively) were synthetically produced with N-terminal fluorescein and C-terminal amidation to mimic their natural charge state. Both were confirmed by circular dichroism to exhibit the characteristic α-helical character in solution (Fig. S5). In pairwise experiments, we equilibrated the peptides individually with AF^680^-labeled shell proteins (PduABB′J) prior to imaging by confocal microscopy. This revealed the strong colocalization of PduD^EP^ and PduP^EP^ with PduB but not PduB′ (Fig. 6A and B). From this, we speculate that the N-terminal portion of PduB may facilitate inward-facing cargo interactions. This is consistent with recent cross-linking mass spectrometry experimentation, which provided direct evidence for interactions between PduB and numerous EP-containing cargo proteins in a native context, as well as other data suggesting that PduB is a shell-cargo anchor point (27, 38). PduA nanotubes were similarly found to colocalize with PduD^EP^ and PduP^EP^ (Fig. 6A). In contrast, colocalization with PduJ was observable but comparatively reduced (Fig. 6D). EPs did not appear to qualitatively influence shell protein morphologies and did not form structures on their own under the conditions used for our experimentation. Furthermore, control experiments with fluorescently modified bovine serum albumin (BSA) showed that it could not appreciably colocalize with PduA nanotubes, supporting the specificity of the EP interactions (Fig. S5). Together, these data tentatively support interactions between EPs and the N terminus of PduB and between EPs and PduA. These data are consistent with the hypothesis that PduB (38, 39) and PduA are likely to be primary factors in facilitating shell-cargo interactions.

## DISCUSSION

**FIG 7:**
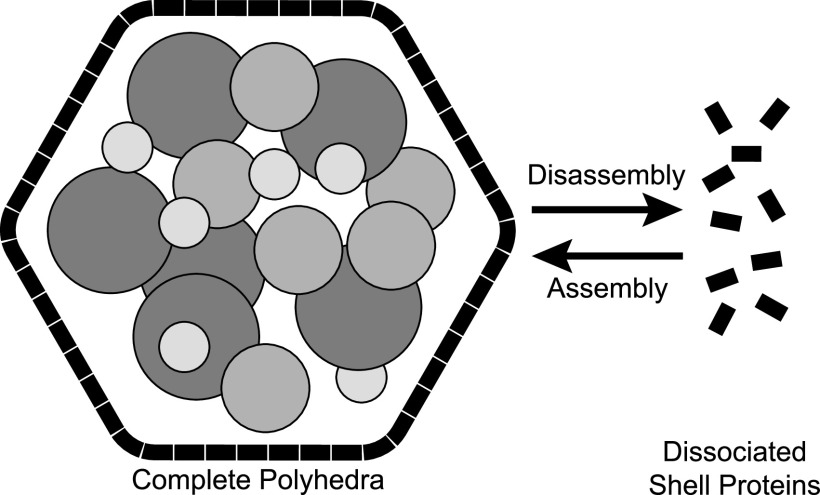
Model for the behavior of the Pdu BMC. Metabolosomes, like the Pdu BMC studied here, may be capable of subunit exchange between complete polyhedra and dissociated shell proteins in the cytosol/solution. This equilibrium is likely to be dictated by the components, the relative and absolute concentrations of those components, pH, salts, and temperature.

In total, the data presented here suggest that confocal microscopy can be used to investigate the exchange of subunits between shell protein superstructures and between fully formed BMCs. Here, we imagine the Pdu BMC as being in an equilibrium between complete Pdu BMCs and individual shell elements in solution (Fig. 7). Our observational timescale is equivalent to the initial stages of procarboxysome formation observed in *Synechococcus* sp. PCC 7942 (40), where one would expect ample protein intermixing prior to budding. Interestingly, previously reported atomic force microscopy experiments showed that BMC-H sheets assemble rapidly and can exchange subunits (41, 42); perhaps, BMC polyhedron facets behave similarly.

We speculate that large, native BMC shells may be able to participate in subunit exchange with “loose” shell proteins in solution (Fig. 7). While we present data showing the colocalization of separately labeled Pdu BMCs and their individual components, inferences from the literature may further tentatively support this notion. For instance, the Pdu BMC will permit the passage of relatively large chemicals (27). While the inner metabolism of some BMCs may create toxic aldehydes that the shell sequesters (43, 44), this does not appear to be a necessity as these metabolic pathways are sometimes encoded without the need for shell proteins (45). Furthermore, bacteria that encode multiple different BMCs heavily regulate them to prevent their intermixing (46). Recent data also demonstrate that preformed carboxysomes can dynamically remodel their shell *in vivo* (47). It may be the case that BMCs are not strictly rigid in order to be more responsive to environmental changes, perhaps so that they can be rapidly assembled and disassembled based on the cell’s metabolic needs. We would add that while BMCs may form through phase separation (48), there is little evidence to suggest that the shell itself is fluid, particularly in an assembled state, and our model does not necessitate that. Still, the shell layer of BMCs may exhibit a high-enough degree of protein exchange that it can be measured experimentally and may therefore be reflected in the colocalization of BMC proteins observed in this study. However, the data presented here are a largely qualitative assessment of this phenomenon. It should be noted, however, that the recent modeling of BMC shell assembly around fluid condensates argues against an interchangeable shell for idealized systems much smaller than typical BMCs (49). It is also possible that the structural features that we observe in this study may exhibit qualitative differences from the structures formed within bacterial cells. Therefore, more evidence, particularly from *in vivo* systems, is needed to clarify the role of BMC protein intermixing. Indeed, an enhanced understanding of BMC dynamism would strengthen how we view the BMC life cycle from biogenesis to degradation.

The fact that the PduA and PduJ structures display differences calls into question the assumption that PduA and PduJ are functionally redundant for shell assembly. Both have been noted to form nanotubes (typically in bundles) and sheets *in vitro* and *in vivo*, to the extent of interfering with septation (23), as have other BMC-H proteins (22, 24). Within an icosahedral context, a recent report suggested that the PduB trimer specifically accommodated PduA and PduJ subunits along different interaction interfaces (27). Together, these data suggest that the assembly of the shell layer might be affected by subtle differences in BMC-H proteins, which are particularly widespread among BMC operons (3). While not investigated here, we note that conditions such as temperature and, in particular, pH and salt may also influence the morphologies of these structures. Elucidating these subtle differences among BMC-H proteins will help future efforts to design novel BMC shells of various sizes and packaging capacities.

Colocalization analysis also revealed that several Pdu EPs interacted specifically with the shell proteins PduAB but did not appear to associate with PduB′J under our assay conditions. Curiously, PduAB are also encoded by the first two genes in the S. enterica *pdu* operon (50). This observation may be consistent with the speculation that PduAB may colocalize with cargo to an EP-driven prometabolosome in the initial stages of biogenesis coupling the shell and cargo. PduAB could then seed further shell envelopment and eventually lead to mature BMCs. This model is analogous to the concomitant assembly proposed for α-carboxysomes (1, 51) despite metabolosomes being more related to β-carboxysomes (52). We speculate that a liquid-liquid-phase separation (LLPS) mechanism is likely to apply to the early stages of metabolosome assembly, as supported by recent reports focusing on the Pdu BMC (53). This has also been suggested for both α- and β-carboxysomes, supporting the notion that LLPS may be a common mechanism for BMC biogenesis (54, 55). Our data do not support the shell or shell protein structures themselves being liquid. In conjunction with other reports that focus mainly on the condensation of cargo, perhaps the shell participates in early BMC biogenesis but then acts to constrain the inner coacervate in a mature BMC.

Confocal microscopy is a powerful tool to study both *in vitro* and *in vivo* colocalization events. Here, we leveraged its ability to differentiate protein components to begin to understand how BMC shell protein structures and whole BMC polyhedra interact in solution. This experimental approach complements traditional TEM approaches. We anticipate that the experimental conditions for laser scanning confocal microscopy can be varied extensively to ask many different questions about BMCs or the superstructures formed by BMC shell proteins. For example, future studies may wish to go further to include time-lapsed measurements or to incorporate emerging superresolution microscopy techniques or fluorescence correlation spectroscopy (56). Therefore, we expect that confocal microscopy will continue to serve as an important tool for investigating shell protein nanotubes in solution, as we showcase here, as well as how they might operate as synthetic *in vivo* scaffolds or if developed as potential biomaterials.

## MATERIALS AND METHODS

### Purification of shell proteins.

All shell proteins were purified under the same set of conditions. Vectors encoding the shell proteins PduABB′JN were transformed into Escherichia coli T7 Express cells. For protein production, strains were streaked from glycerol stocks onto LB agar with carbenicillin. Single colonies were then used to produce overnight starter cultures. The next day, starter cultures were used to inoculate 500 mL of 2× YT broth supplemented with carbenicillin and grown to an optical density at 600 nm (OD_600_) of 0.6 to 0.8. Once the cultures were at the appropriate density, isopropyl-β-d-thiogalactopyranoside (IPTG) was added to 0.5 mM to induce protein expression. Cultures were further incubated at 37°C for 3 h before being pelleted and frozen at −80°C. Pellets were then thawed and resuspended in 40 mL buffer A (50 mM Tris-HCl [pH 8.0], 1 M NaCl, 5 mM dithiothreitol [DTT], 10 mM imidazole) supplemented with 5 mM MgCl_2_, 1 U/mL baseline-zero DNase (Lucigen), and 0.5 mM phenylmethylsulfonyl fluoride (PMSF). Cells were then lysed by sonication, and insoluble matter was pelleted by centrifugation at 13,000 × *g* for 20 min. Soluble matter was then incubated with 1 mL of nickel-nitrilotriacetic acid (NTA) affinity beads that had been preequilibrated in buffer A for 30 min on ice. The flowthrough was drained, and beads were washed with 5 mL of buffer A followed by 5 mL of buffer B (50 mM Tris-HCl [pH 7.4], 1 M NaCl, 35 mM imidazole). This second wash removes nonspecifically bound proteins and equilibrates bound samples for fluorescence labeling. The appropriate fluorophore (Alexa Fluor maleimide 488 or 680) was dissolved in 1 mL of buffer B and added to the bead bed, where the reaction was allowed to proceed for 2 h at 4°C. The excess fluorophore was then drained off, and the beads were washed again twice with 5 mL of buffer B supplemented with 5 mM DTT. Labeled proteins were then eluted in buffer C (50 mM Tris-HCl [pH 8.0], 1 M NaCl, 5 mM DTT, 250 mM imidazole) and dialyzed into storage buffer (20 mM Tris-HCl [pH 8.0], 100 mM NaCl, 5 mM DTT). Aliquots were dispensed and flash-frozen for future use.

### Pdu BMC purification from a heterologous host.

S. enterica Pdu BMCs were expressed and purified from an E. coli host R995+PduST (57). Briefly, A single colony of R995+PduST was used to inoculate 100 mL of 2× YT supplemented with kanamycin and 0.5% 1,2-propanediol and grown overnight at 37°C for ~16 h. The next day, the culture was pelleted; resuspended in 20 mL of a solution containing 50 mM Tris-HCl (pH 8.0), 200 mM KCl, 5 mM MgCl_2_, 5 mM β-mercaptoethanol (βME), 0.5 mM EDTA, 0.5 mg/mL lysozyme, 0.5 mM phenylmethylsulfonyl fluoride (PMSF), and 50% (vol/vol) B-PER (Thermo); gently rocked at room temperature (RT) for 25 min; and then placed on ice for 5 min. Genomic DNA was fragmented by two 1-s pulses from a sonicator. Insoluble matter was pelleted for 20 min at 13,000 × *g* at 4°C. BMCs were pelleted at 17,500 × *g* at 4°C for 40 min. Pelleted BMCs were then resuspended in labeling buffer (20 mM phosphate [pH 7.4], 50 mM KCl, 5 mM MgCl_2_) and pelleted again for 20 min. The final BMC pellet was resuspended in 1 mL of labeling buffer and stored at 4°C for <2 weeks. BMCs were quantified via a Bradford assay (58) against a BSA standard.

### Preparation of Pdu BMCs for confocal imaging.

The preparation of Pdu BMCs for confocal imaging is outlined in Fig. S4 in the supplemental material. Briefly, purified Pdu BMCs were diluted to 1.0 mg/mL in labeling buffer (20 mM phosphate [pH 7.4], 50 mM KCl, 5 mM MgCl_2_) and labeled with 125 μM fluorophore for 15 min on ice. The reaction was quenched with 5 mM DTT. Pdu BMCs were then pelleted at 200,00 × *g* for 15 min at 4°C and resuspended in fresh labeling buffer to remove all excess fluorophore. When appropriate, Pdu BMCs were then fixed in the presence of 2% paraformaldehyde and 5 mM (PEGylated bis(sulfosuccinimidyl)suberate) (BS(PEG)5) cross-linker for 30 min at room temperature.

### Laser scanning confocal microscopy imaging.

All samples were imaged on a Zeiss LSM 980 Airyscan system at room temperature using a 63× objective with immersion oil. All samples were likewise prepared in labeling buffer (20 mM phosphate [pH 7.4], 50 mM KCl, 5 mM MgCl_2_) for all experiments. Briefly, 20 μL of the sample solution (typically 20 μM protein) was pipetted directly onto a number 1.5 coverslip (catalog number 12544E; Fisherbrand). Initial focus was achieved by focusing on the edge of the sample droplet under transmission mode before switching to fluorescence and focusing down to the coverslip surface. Structures are plentiful and bright enough to allow live calibration of the Airyscan detector in superresolution mode. The fastest possible image scan time was always used, with a minimum pixel scan time of 1.00 μs. All captured and reported images had standard Airyscan processing applied. Captured, unedited images were all analyzed using ImageJ. The JACoP ImageJ plug-in was used to facilitate van Steensel cross-correlation analysis (34).

### Transmission electron microscopy.

Samples were thawed and diluted in labeling buffer (20 mM sodium phosphate [pH 7.4], 50 mM KCl, 5 mM MgCl_2_) to 20 μM, and 5 μL was applied to a Formvar-coated 200-mesh copper grid (catalog number FCF200-CU; Electron Microscopy Sciences). Samples were air dried for 15 min, and the excess solution was wicked off. Salt was removed by three 5-μL additions of deionized water before staining with 3 μL of 2% uranyl acetate for 10 s. Samples were imaged using a JEOL 100CXII electron microscope.

### Circular dichroism of EPs.

Synthetically derived EPs were purchased from GenScript. Dried peptides were dissolved in ice-cold deionized water to a concentration of 500 μM and stored on ice until analysis. For analysis, peptides were diluted to 50 μM in deionized water in a 1-mm quartz cuvette. Measurements were taken on a Jasco J-810 circular dichroism spectrometer from 180 to 300 nm in 1-nm increments with a scan speed of 100 nm/min in triplicate, with moderate smoothing.

### EP-shell protein binding assay.

Synthetic EPs were resuspended in 10 mM Tris-HCl (pH 8.0). After EP resuspension, EPs and shell proteins were mixed (4 μM and 40 μM, respectively) and equilibrated on ice for 30 min before imaging. All images between samples were taken with the same laser intensity and detector settings.
